# Quality of perinatal depression care in primary care setting in Nigeria

**DOI:** 10.1186/s12913-018-3716-3

**Published:** 2018-11-22

**Authors:** Olatunde O. Ayinde, Bibilola D. Oladeji, Jibril Abdulmalik, Keely Jordan, Lola Kola, Oye Gureje

**Affiliations:** 1 Department of Psychiatry, University College Hospital, Ibadan, PMB 5116 Nigeria; 2 College of Global Public Health, New York University, New York, USA

**Keywords:** Perinatal depression, Chronic care model, Primary care, Low and middle income countries

## Abstract

**Background:**

Even though integrating mental health into maternal and child health (MCH) is widely accepted as a means of closing the treatment gap for maternal mental health conditions in low- and middle-income countries (LMIC), there are not many studies on the quality of the currently available mental health care for mothers in these countries. This study assessed the existing organization of service for maternal mental health, the actual care delivered for perinatal depression, as well as the quality of the care received by affected women presenting to primary care clinics in Ibadan, Nigeria.

**Methods:**

The Assessment of Chronic Illness Care (ACIC) tool was administered to the staff in 23 primary maternal care clinics and key informant interviews were conducted with 20 facility managers to explore organizational and administrative features relevant to the delivery of maternal mental health care in the facilities. Detection rate of perinatal depression by maternal care providers was assessed by determining the proportion of depressed antenatal women identified by the providers. The women were then followed up from the antenatal period up until 6 months after childbirth to track their experience with care received.

**Results:**

All the facilities had ACIC domain scores indicating poor capacity to offer quality chronic care. Emerging themes from the interviews included severe manpower shortage and absence of administrative and clinical support for manpower training and care provision. Only 31 of the 218 depressed women had been identified by the maternal care providers as having a psychological problem throughout the follow-up period. In spite of the objective evidence of inadequate care, most of the perinatal women rated the service provided in the facilities as being of good quality (96%) and reported being satisfied with the care received (98%).

**Conclusion:**

There are major inadequacies in the organisational and administrative profile of these primary maternal care facilities that militate against the provision of quality chronic care. These inadequacies translate to a large treatment gap for women with perinatal depression. Lack of awareness by service users of what constitutes good quality care, indicative of low service expectation, may hamper user-driven demand for quality improvement.

## Background

Depression is the leading cause of disease burden in women of reproductive age [1]. Perinatal depression affects up to 25% of women in LMIC in the antenatal period and 19% in the postnatal period, rates that are significantly higher than reported for high-income countries [2]. In Nigeria, studies have reported rates of up to 10–30% amongst perinatal women [3–7]. Apart from the suffering and loss of productivity that result from this disabling condition [8], perinatal depression is an important risk for suicide, a public health problem that is expected to become the leading cause of maternal mortality in LMIC [9]. Perinatal depression is also associated with short and long term adverse maternal and child outcomes, such as pre-term birth and low birth weight, poor mother-child interactions, infant under-nutrition and stunting, higher rates of diarrhoeal diseases, poor infant development, poor interpersonal functioning, insecure attachment and higher rates of emotional and behavioural problems in infants of depressed mothers [10, 11]. There are indications that these adverse child outcomes are worse in LMIC [10]. There is also evidence for the intergenerational transmission of the socio-economic disadvantages associated with perinatal depression, such that its effects continue to echo in the lives of offspring of affected persons up to 18 years after birth [12]. In view of the foregoing, there has been a global call for the expansion of services for perinatal depression globally and especially in LMIC. However, there is evidence suggesting that a focus on coverage alone without due attention to quality of care provided might have hampered the achievement of both the development goals and the protection of human rights of mothers and children in LMIC during the Millennium Development Goals (MDG) era [13].

Perinatal women living in LMIC are at an elevated risk of being denied quality care. The majority of such women have access only to facilities that are poorly resourced [14, 15]. There is an additional disadvantage for women with perinatal depression that is attributable to the pervasive stigma associated with mental illness, especially in LMIC. Persons with mental disorders are often victims of negative attitude that manifests in the form of discrimination and denial of basic rights [16]. Such persons may also internalize shame, anticipate rejection and discrimination, and accept diminished expectations from others [17]. These two forms of stigma, enacted and felt, have the effect of exposing persons with mental disorders to poor and inequitable quality care [18]. The situation is complicated by the pervasive lack of awareness of the true medical nature of depression as well as diverse attributions offered by affected persons that may limit appropriate medical help-seeking [19]. In the context of perinatal depression, therefore, stigma would increase the likelihood of sufferers to being denied access to the basic and oftentimes rudimentary services that may be on offer.

Addressing quality concerns in maternal health, as in any other area of health, requires an examination of both micro-level factors such as the structure of clinics and provider behaviour, as well as system-wide factors such as the design of service delivery systems and governance structures [20]. The Lancet Global Health Commission [20] defines a high quality health system as one that “optimizes health in a given context by consistently delivering care that improves or maintains health, by being valued and trusted by all people, and by responding to changing population needs” [20]. The Institute of Medicine recognises six domains of quality, namely, safety, effectiveness, patient centeredness, timeliness, efficiency, and equitable care) [21]. For many patients, depression occurs in the context of other medical conditions and requires long term care. Hence, for depressed patient to receive improved quality care and have better health outcomes, it has been argued that they be managed within an integrated chronic disease model in primary care [22, 23], where the bulk of them presents. The Chronic care model (CCM) [24] is one of the well-known integrated care models that have been extensively used in the management of depression in primary care. The core concept in the CCM includes a system design that ensures a well-coordinated team with clearly defined roles for each team member, delivering evidence based interventions, with the client playing an increasing role in her own care; well-planned provider-client interactions, with regular follow up; information system that facilitate patient tracking and linkage with community resources through partnerships. Elements of the CCM embedded in routine care have been shown to produce better outcomes for mental disorders, and depression specifically, in primary care setting, both in high income countries and LMICs [22, 25]. Perinatal depression managed as a chronic condition within an integrated maternal care setting would therefore approximate a standard of quality care, against which a test model of care can be compared.

In order to understand the capacity of maternal primary care clinics in Nigeria to deliver care to those in need of service, this study aims to examine the existing organisational structures of the selected facilities as well as the current level of care provided to women with perinatal depression living in poor social and economic circumstances. In this study, we sought to address three questions: 1) What is the current organizational structure of maternal clinics in regard to the delivery of quality chronic care? 2) How able are providers in these clinics to identify and provide treatment for women with perinatal depression throughout the antenatal period and up to 6 months postnatal period? And 3) How do women with perinatal depression rate the standard of care in the clinics?

## Methods

We conducted a formative study as a part of an implementation research project to assess the factors that may promote or hinder the delivery of quality service to women with perinatal depression. The project, Scaling up Care for Perinatal Depression for Improved Maternal and Infant Health (SPECTRA), is being conducted in 23 randomly selected primary health care (PHC) facilities across all the 11 local government areas (LGAs) in and around the metropolitan city of Ibadan in the south-western part of Nigeria. The clinics were selected based on the sample size calculated for the number of depressed women required for the parent study, (details are available from authors on request).

In addressing our research questions, a mixed methods approach was employed. For the first question, we administered the Assessment of Chronic Illness Care (ACIC) [26] to the facility managers in 23 maternal care clinics. The ACIC is a widely used tool to evaluate the standard of care provided to patients who are in need of sustained care [26, 27]. Specifically, we assessed the capacity of the maternal care facilities to provide care and support to patients with perinatal depression. The items of the tool, which are organised into 7 domains (organisation of the healthcare delivery system, community linkages, patient support for self-management, decision support for service providers, delivery system design, clinical information system, integration of chronic care components) are scored on a four-point Likert scale, giving an average domain score that ranges from 0 to 11, as well as a Total Programme Score. A score between “0” and “2” corresponds to the lowest level of support for chronic care; between “3” and “5”, basic support, between “6” and “8”, reasonably good support while a score between “9” and “11” indicate fully developed chronic illness care programme.

Key informant interviews (KIIs) were conducted with the facility managers of 20 of the 23 the selected maternal clinics (3 were not available for study) to enquire about the process of care for perinatal women, the organizational and administrative features of the clinics that may have a bearing on the care of women with perinatal depression, including those for training and supervision of the primary health care workers (PHCWs). The facility managers were experienced midwives, community health officers or community health extension workers who had intimate knowledge of the clinical and administrative environment of the facilities and of the government departments that regulate them, and who had risen through the ranks to become facility managers. Basic nursing education in Nigeria is a 3-year diploma programme, which can be further supplemented by an additional 18-month programme to earn another diploma in midwifery. The Community Health Officers and Community Health Extension Workers spend 2 and 3 years post-secondary school respectively to earn clinical practice diplomas suitable for working in primary care. The interviews were conducted in English language by female research assistants with a minimum of Master’s degree in the social sciences and with prior experience in qualitative interviews. Prior to the interviews, the research assistants established contact with the participants, explained the objective of the study to them and obtained their consent. The interviews were audio-recorded and field notes were taken to supplement the audio recordings. The interviews were one-off, face-to-face interviews, conducted at the respective maternal care clinics of the participants, according to an interview guide developed from the literature on integrated chronic depression care and drawing on our previous experience of such assessment [19]. Each interview took between 45 min and one h.

To address the second question, consecutively registered women presenting for routine antenatal care and who had previously consented to participate in the study were screened with the Edinburgh Postnatal Depression Scale (EPDS) [28] to identify those with perinatal depression, after they had been seen by the PHCWs. The EPDS has been validated in our setting and, for the purpose of this exercise, with a score of 10 or more out of a possible total of 30 shown to be indicative of moderate depression [29]. Over a period of 6 months, 218 women who screened positive on the EPDS were identified as cases of depression. We made a determination of whether a woman who screened positive to depression using the EPDS had been recognised as having a psychological problem by the maternal care provider. For this, we used a fairly broad definition of detection. First, we reviewed the case records made on each of the patients by the providers to see whether any entry had been made to indicate that a psychological problem had been noted or treated at baseline. That is, the entry did not have to specify a diagnosis, just an indication of any symptoms or reported emotional problem. Second, we conducted an assessment of the women who screened positive in their homes within 72 h of being screened in the maternal care clinic (baseline assessment). We enquired from them whether they had been asked any question about their mood, sleep, worry or any other emotional concern. We repeated this assessment at the women’s home at 2 months post recruitment, at delivery, 3 months postpartum and 6 months postpartum. Any screen-positive woman whose case record showed an entry of possible emotional problem or who, at interview, reported having been offered any form of counselling was deemed to have had their depression detected by the maternal care provider.

To address the third question, irrespective of the answer they provided to the questions above, the respondents were also asked, during the 6-month postnatal follow-up, to rate 1) the overall quality of care provided in the clinics 2) their level of satisfaction with care provided during both antenatal and postnatal clinic visits and 3) how accessible, acceptable and convenient these services were. Each of the items was framed in such a way that it can be rated on a binary “Yes/No” scale.

### Data analysis

The KIIs were transcribed verbatim by the research assistants who conducted the interviews. OOA read each transcript and assigned codes manually after familiarizing himself with the transcribed data. The codes were then organised into themes that had been identified beforehand from relevant literature. BDI reviewed and cross-checked the codes and themes originating from them. Any identified discordance was discussed and final findings from the themes represents consensus between the two. In presenting the findings, direct quotes are provided where appropriate.

Quantitative data were analysed with SPSS version 25. Summary statistics and simple frequencies were used to display continuous variables and proportions respectively.

## Results

### Assessment of chronic illness care (ACIC)

All 23 facilities sampled had ACIC scores that correspond to the lowest level of institutional support for chronic care across five domains (organisation of the healthcare delivery system, patient support for self-management, decision support for service providers, clinical information system and integration of chronic care components) as well as on the total domain score and only two facilities had scores corresponding to basic level of support for chronic care across two ACIC domains (community linkages and delivery system design). None of the facilities had scores reflecting reasonably good support for chronic care or a fully developed chronic care for depression in any ACIC domain.

### Key informant interviews with facility managers

#### Demographic characteristics and perceived competence to respond to perinatal depression

The facility managers interviewed (*N* = 20) were predominantly females (95%), were all senior midwives, except two, one of whom was a senior community health officer while the other was a community health extension worker. Their mean age was 48.7 ± 7.71 years and mean number of years of experience was 24.4 ± 5.37 years.

All the facility managers claimed they had received training on mental health, in general, and on depression, specifically, as students for periods lasting between 3 weeks and 6 months. Some of them had also received additional on-the-job training from mental health professionals in the context of previous research in the course of their careers. Facility managers who spent longer periods on these trainings and had participated in more of the in-service training expressed more confidence in the adequacy of their training to respond to perinatal depression while most of those who spent less time in training expressed some doubt about their ability to manage perinatal depression. The most frequently mentioned modern modes of treating perinatal depression by the managers were counselling and the use of medications. Less frequently mentioned approaches included “problem solving” (one respondent), “empowerment” (one respondent), “psychoeducation” (two respondents), “psychotherapy (three respondents)” and “blood pressure management” (one respondent).

One manager, a senior mid-wife with 25 years clinical experience, felt very well trained and confident about her training and ability to manage perinatal depression.

*“…you know the training in University College Hospital now… when we were having our training there... you would be thinking like you are going to become a doctor, because there would be a lecturer to teach you in class and they would invite a psychiatrist to come and teach again… they would post you to psychiatry ward to go and work there and then we still went to Abeokuta Aro Mental Hospital. So with all these experiences, we can’t forget, because we were taught… let me say for a total of 5 months at least…”.*

A Community Health Officer who did not exude much confidence about her capacity said:

*“We were taught in school… they taught us about depression but we were not taught deeply….when we did Community Health Officer (course)… they taught us about depression and psychosis, but it is not so deep…. For just about 3 months.”*

#### Facility arrangement

All facilities were managed by an experienced midwife/nurse, senior community health officer (CHO) or senior community health extension worker (CHEW). The frontline care providers in the clinics were nurses, CHOs and CHEWs. The latter two cadres had between 2 to 3 years of post-secondary school clinical training. Occasionally, a medical doctor visited the clinics, if the local government area under whose jurisdiction the clinic was had a medical officer of health in its employ. Only one of the facility heads in this cohort reported having a medical doctor in attendance in clinic on some weekdays. The clinicians worked in shifts and in different sections of the clinics, such as those for immunization, antenatal care and for minor injuries. All the facility managers complained about severe shortage of manpower, which some of them addressed by employing ad hoc staff who might not be on the general payroll but were remunerated from funds generated internally at the facility level. In one of the facilities, ad hoc staff made up four of every five of the total staff strength. One manager captured this as “too few workers being engaged in too many tasks”. A 51 year old Chief Nursing Officer said: “our staffs (sic) are not enough. (There is) no one who doesn’t know that staffs (sic) are insufficient everywhere. Not just here… but since it is the work that we do, we cannot leave it undone…”.

#### Process of care and structural features of the clinics that may have a bearing on the care women with perinatal depression receive

Concerning facility level arrangement for the management of chronic conditions, the facility managers identified tuberculosis, HIV, diabetes mellitus and hypertension as chronic illnesses for which some kind of chronic care should be instituted. Two of them mentioned mental illnesses such as substance addiction, but not depression, as chronic illnesses. The facility managers claimed that there were organizational arrangements for chronic disease management for HIV and Tuberculosis. Frequently cited arrangements included management guidelines for tuberculosis and HIV, commonly provided by funding agencies and government ministries of health. These protocols usually specify the procedures for diagnosis, treatment (including assertive follow-up for treatment adherence) and referral. No specific chronic care arrangement was identified for perinatal depression.

Although the facility managers were aware that personalised care was an important part of chronic care, only a few of them ensured that the same care providers followed up clients in need of chronic care. Two of the managers claimed they did this for patients with perinatal depression because they had been taught in a previous project that it was the way to provide effective care for women with perinatal depression. When patients did not show up for follow up, frequently employed method of reaching them included contacting them through relatives and friends who were receiving care at the same clinics, use of mobile phones as well as home visits when feasible. The facility managers however admitted that phone calls were paid for out of their own pockets, which was unsustainable and home visits were rare and difficult because of extreme shortage of human resources. Most of them reported that their clinics did not have special appointments for chronic care patients.

The majority of the facility heads judged the current care arrangement for perinatal depression at their clinics as “adequate”. A few however disagreed. A Chief Nursing Officer who had been on the job for 24 years claimed that the care arrangement for depression in her clinic was “not adequate because of non-availability of drugs”. “I know how to counsel them but I don’t know how to administer drugs”. Some of those who claimed that the current care arrangement was adequate admitted that they judged the current care arrangement adequate because they could refer cases of perinatal depression to higher levels of care.

A 50 year old Senior Community Health Extension Worker with 30 years’ experience said:

*“The reason for being (sic) adequate is that we don’t stress them too much before we refer them to Adeoyo or UCH (secondary and tertiary centres respectively) because most people that come here are low cadre people. Here is a local government, a primary health care centre, so with the treatment we give them, it is adequate.”*

#### Administrative environment for training and supervision of PHCWs

In regard to supervision in the clinics, majority of the facility managers claimed that some form of general supervision existed from facility level up to the federal ministry of health. A chief nursing officer of 24 years said:

*“…Right now, most of us in the morning (shift) do most of the supervision. When the people on morning duty leave, the works (*sic*) that people in the afternoon and night shift do, when we resume in the morning, we go through their report and register. For the people on the afternoon shift, if there is any mistake in their work, when they come back to work, we explain to them that we have checked through their work and this is how they (are) supposed to do it, and we would put them through. That is how we do it”.*

She said further:

“…*doctors are the ones in charge of our supervision. For example, today is M&E meeting for monitoring and evaluation. Doctors and MCH (maternal and child health officers)... That is how they used (sic) to do it.”*

All the facility managers claimed that there was no organisational support for training of members of staff for perinatal depression at facility, local, state or federal government level. A 43 -year old community health officer complained:

*“There is no administrative support for training. There is none. There is no provision for it. There is nothing for pregnant women with depression from government. They have not talked about it before. There is nothing. There is none at all”*.

The facility level and organisational difficulties encountered in responding to perinatal depression that were enumerated by the facility managers included severe shortage of manpower, non-availability of medications, irregular and non-payment of staff remunerations, inadequate facility space for psychosocial interventions and absence of continuing training for members of staff. Recommendations for improvement of chronic care for perinatal depression included consistent recruitment of healthcare workers, training of providers in primary care clinics, and the establishment and training of a cadre of provider-counsellors as “specialist providers” dedicated to treating perinatal depression.

### Screening and baseline demographic and treatment characteristics of perinatal women

Over a period of 18 months, a total of 2989 consecutively registered antenatal women were screened with the EPDS of which 218 (7.3%) screened positive (that is, scoring 10 or more). The mean age of the women who screened positive was 25 ± 6.2 years and the mean gestational age was 23 ± 4.2 weeks. The mean EPDS score at screening was 12 ± 2.4. Other demographic variables are shown in Table 1. Of the 218 clients who screened positive for perinatal depression, only three (1.4%) were identified by PHCWs as having the condition as indicated in their case records at first clinic contact.

**Table 1:** Socio-demographic profile of care recipients at the PHC clinics

*N* = 218	n	%
Age		
Mean (SD) = 25 (6.2) years		
Years of education		
Mean (SD) = 11 (2.8) years		
Marital status		
Single /widowed	52	23.9
Married /Cohabiting	166	76.1
Parity		
0	106	48.6
1 or more	112	51.4
Occupation		
Professionals and non-manual workers	12	5.5
Unemployed, manual workers, traders, and artisans	206	94.5

Treatment options offered included diazepam tablet for one participant, counselling for the second one and no treatment was recorded for the third one None of the women was provided with structured psychosocial intervention or offered follow-up specifically to address their depression.

When interviewed in their homes by research assistants about their clinic experience, 13 (6.3%) of the women indicated that they were asked specifically about mental health symptoms and signs by the maternal care providers. The commonest elicited symptom was that of difficulty with sleep. Other less common symptoms enquired about included sadness, suicidal behaviour and “excessive thinking”. Two of the three patients with recorded evidence of detection of their depression claimed they were not asked about mental health symptoms when interviewed 72 h later, but were nevertheless recorded in the clinical notes as being depressed. Of the 13 women asked about mental health symptoms at baseline, only one was asked about these symptoms again at follow up.

Most of the women (96.1%) assessed the quality of care provided in the clinics to be good and 98% reported that they were satisfied with the care they had received on their first clinic visit. Of the 205 respondents, 11 (5.4%) reported some difficulty in the physical accessibility of the PHC while 18 (8.8%) reported that the fees charged for treatment and medications were unaffordable. Five of the women (2.4%) claimed that seeking treatment at the PHC made them feel embarrassed or ashamed.

### Follow up care

At the 2 month post-recruitment assessment, only 164 of the recruited 218 perinatal women were available and/or eligible for assessment. By this time, an additional 16 women (9.6%) reported having been asked about mental health symptoms at subsequent visits to the clinics (Table 2).

**Table 2:** Care received for perinatal depression by women presenting at antenatal clinics

	Screening	Baseline (within 72 h of screening)	2 months post recruitment	3 months postpartum	6 months postpartum
Eligible/available for assessment	218	205	164	173	155
Detected or asked about mental health symptoms (n, %)	3 (1.4)	13 (6.3%)	16 (9.8)	2 (1.2)	Nil
Offered counseling (n, %)	2 (0.9)	Not applicable	16 (9.8)	Nil	Nil
Recovery from depression	Not applicable	Not applicable	88 (53.7)	110 (63.6)	93 (60.0)

All 16 women claimed they were offered some counselling. At the end of the second month following recruitment, a total of 29 of the antenatal women had either case record entry of a diagnosis of depression or of a psychological distress or reported having been asked about symptoms of depression or of psychological illness broadly defined, and 28 of them had been offered some form of counselling. The proportion of women who claimed they were satisfied with the treatment they had received at the 2 month assessment was 93.7%.

At the 3 month postnatal assessment, two more women had been asked about mental health symptoms by the PHCW. In total, at the end 6-month postpartum follow-up, only 31 of the women available for assessment could be said to have either been diagnosed with a psychological problem or indeed to have been asked questions specifically indicating that such a problem might have been suspected by the PHCW. Of these 31, there was suggestive evidence that 28 had been offered some form of counselling.

Recovery from perinatal depression defined as EPDS score of less than 6, had occurred in 88 of the 164 (53.7%) women assessed at 2 months post recruitment. At 3 months postpartum, of the 173 women available for assessment, 110 (63.6%) had recovered from perinatal depression. At the 6 month assessment, 60% of the 155 women available for assessment had recovered from depression.

Table 3 shows details of the obstetrics outcome of the perinatal women at the end of the follow up period. Almost a quarter of women delivered their babies in their own homes. About 10% had either assisted delivery or Caesarean section. Three births were twins and the rest were singletons. Other than three reported still births, immediate obstetric outcomes were generally favourable.

**Table 3:** Obstetrics and Infant outcomes (*N* = 188)

	Number	Percent
Place of delivery		
Clinic	121	64.4
Home	44	23.4
Mission Home	23	12.2
Mode of delivery		
Normal vertex	170	90.4
Assisted	6	3.2
Cesarean section	12	6.4
Nature of birth		
Singleton	185	98.4
Multiple birth	3	1.6
Status of infant		
Live	185	98.4
Stillbirth	3	1.6

## Discussion

In this study we explored the process of care for women with perinatal depression as well as the structural and organisational features of PHC clinics that may have a bearing on the provision of good quality care for perinatal women in Nigeria. We also documented the quality of care received by these women for this condition starting from their antenatal period to 6 months postpartum.

The facility managers in this study identified severe shortage of frontline PHCWs in the clinics, irregular payment of workers’ salaries and lack of regular on-going in-service training for providers on how to respond to perinatal depression. It is generally agreed that the most feasible way of scaling up care for perinatal conditions is through the horizontal integration of mental health into primary and maternal health care. At the heart of this is the task sharing approach that involves non-specialist health workers, who may also be non-physicians, delivering most of the care while specialists provide training, supervision and support as well as care for the most severe cases. A fundamental assumption of this model is that the PHCWs are adequate in number and are well remunerated and motivated to provide service. Our finding corroborates an earlier report of a survey of PHC clinics in four states in Nigeria where, in addition to understaffing across all states, urban-rural disparities in the distribution of PHCWs were found [30]. Integration of mental health into primary care presupposes a fully functional PHC system with the full staff compliment. Severe shortage of health manpower is a common feature of most countries in Sub-Saharan Africa, and this makes it imperative for policy makers to institute strategies that are aimed at attracting and retaining health professionals especially in primary care which makes up the bulk of the health systems in these countries. In Nigeria, for example, the PHC system makes up 85% of the health system [31] and is the level of care used the most by the poor.

The results from the qualitative and quantitative aspects of this study show that organisational and facility arrangement for chronic care generally and perinatal depression, in particular, was inadequate. Among the facility level factors that may hinder provision of quality care for depression as identified by managers include an absence of a clear-cut protocol for managing perinatal depression at the facilities, difficulties with scheduling follow up with the same providers, absence of administrative support for training and of clinical support for the management of perinatal depression. These conditions at the clinics fall short of the requirements of the components of CCM for delivering good quality service for perinatal depression. Components of the CCM require evidence based guidelines incorporated into daily practice, continued productive interactions between informed clients who take active parts in their own care and their care teams or providers, which is ensured by an efficient follow up system with the same provider [32].

Compared to the detection rates reported from previous research in better resourced health systems, the detection rate found in our study was very low. In the United Kingdom, a combined team of general practitioners, midwives and health visitors identified about half of the cases of perinatal depression, although it took an average of 14 contacts with the clients to detect them [33]. Similarly primary care physicians in Chile were able to detect 48% of cases of common mental disorders presenting in primary care [34]. However, the detection rate found in the current study compares well with detection rates of mental disorders or depression in primary care reported in other LMICs, which range from 0 to 1.3% [35–37]. The low detection rates of depression among PHCWs in LMICs have been attributed to low level of mental health knowledge and skill among PHCWs, and the prioritization of physical conditions over mental disorders when depressed patients present with multiple somatic symptoms [35]. The low capacity of the sampled facilities to provide quality chronic care for depression and the low detection rates for depression by the PHCWs demonstrate important gaps in both the organisational structures and manpower capacity of the frontline facilities to respond to common perinatal mental health conditions in a fully functional integrated chronic care model.

However, beyond the low detection rate of perinatal depression, our finding builds on previous evidence which suggests that capacity to respond to common perinatal mental disorders is virtually non-existent in primary care setting in Nigeria [38], as it is in many LMIC. Our sample consisted mainly of low income, poorly educated women whose only contact with the health care system may be the antenatal clinic. Taken within the broader context of regional and within country inequalities in access to mental health care characteristic of LMIC [39], antenatal women suffering from depression and their unborn children represent some of the most vulnerable populations, and denying them quality mental health care is an ethical concern. This is worsened by the absence of mental health legislation in many LMIC to protect the human rights of vulnerable populations in terms of access to quality mental health care [40]. Access to quality maternal care is not only an important human right for the mothers, it is also inextricably woven into the rights of the child [13].

The vast majority of the patients claimed that the place of care was accessible, care was affordable, and seeking treatment did not make them feel ashamed. It is reassuring that the clinics in our study continue to deliver on the underlying philosophy of PHC design: socially acceptable and universally accessible health care to individuals and families [41]. This finding strengthens the argument for the appropriateness of primary care as the most fitting setting for maternal mental health care.

Despite the objectively poor quality of service being provided, the women using these facilities still rated them high in regard to quality of care and personal satisfaction with level of service provided. This paradox is an important indicator of the existing inequity in the system: persons who have never experienced good quality service set their expectations low and are happy to receive any service provided, oftentimes happy that a favour is being extended to them by the mere presence of any such service [42, 43]. Their inability to envisage a better service means they do not ask for it and the system is thereby deprived of the otherwise powerful motivation for improvement that users’ demand may constitute, thereby setting up a vicious circle. One reason may reflect the observation that depressed perinatal women may not be aware of the medical nature of their problem, attributing the symptoms to various social and supernatural causes for which medical response might not be indicated [19] or appropriate. It is also possible that the immediate obstetrics outcomes, which are generally satisfactory in this group of mothers may have informed their overall assessment of quality. A recent report by the Lancet Global Health Commission on Health Quality [20] advocates for deliberate efforts at developing active, well informed service user population to drive service quality.

There are limitations within which the results of this study should be interpreted. Firstly, although the EPDS has been validated for use in this environment, we did not conduct a second stage assessment to confirm the diagnosis. Secondly, facility and provider quality assessment employed a mixed method approach but service users’ perception of quality was not as rigorous, with no complementary exploratory interviews to shed more light on user experience. It is possible that supplementing service users’ perception of quality with qualitative methods could have yielded more information on the subject.

## Conclusion

This study found major gaps in the health system governance structures, facility arrangements and provider capacity to provide care for perinatal depression in primary care setting. There is a need for policy makers to pay attention to policies and strategies for ensuring access to quality perinatal depression care at the health system level, redesign and reorganise service delivery for quality care at the facility level and equip providers to deliver perinatal depression care through pre-employment and in-service manpower development. There is also a need to develop active, well informed service-user groups to ensure demand-side driven continuous quality improvement in maternal mental health care. Access to quality perinatal mental health care in LMIC is both an ethical and a developmental issue in the SDG era.

## Abbreviations

ACIC: Assessment of chronic illness care
CCM: Chronic care model
CHEW: Community health extension worker
EPDS: Edinburgh postnatal depression scale
LGA: Local government area
LMIC: Low- and middle-income countries
MCH: Maternal and child health
MDG: Millennium development goals
PHC: Primary health care
PHCW: Primary health care worker
SDG: Sustainable development goals
